# Exploring the influence of behavioural, normative and control beliefs on intentions to adhere to public health guidelines during the COVID-19 pandemic: a qualitative interview based study

**DOI:** 10.1186/s12889-023-15344-0

**Published:** 2023-03-10

**Authors:** Jeanna Parsons Leigh, Stephana Julia Moss, Sara J. Mizen, Emily A. FitzGerald, Rebecca Brundin-Mather, Chloe de Grood, Alexandra Dodds, Henry T. Stelfox, Kirsten M. Fiest

**Affiliations:** 1 Faculty of Health, School of Health Administration, Dalhousie University, Halifax, NS Canada; 2 Department of Critical Care Medicine, Alberta Health Services & University of Calgary, Calgary, AB Canada; 3 Department of Community Health Sciences & O’Brien Institute for Public Health, University of Calgary, Calgary, AB Canada; 4 Department of Psychiatry & Hotchkiss Brain Institute, Cumming School of Medicine, University of Calgary, Calgary, AB Canada

**Keywords:** COVID-19, Pandemic, Public health, Theory of planned behaviour, Interviews

## Abstract

**Background:**

Perceived severity and susceptibility of disease are predictors of individual behaviour during health crises. Little is known about how individual beliefs influence intentions to adhere to public health guidelines during periods of health crises, and how access to and consumption of information influence these intentions. This study investigated behavioural beliefs, normative beliefs, and control beliefs, and their influence on behavioural intentions to adhere to public health guidelines during the COVID-19 pandemic.

**Methods:**

Participants were recruited from a related COVID-19 study conducted by our team, and through snowball sampling in subsequent. Using a maximum variation sampling technique, we recruited a diverse group of participants representing six major regions in Canada. Participants took part in one-on-one semi-structured interviews from February 2021 to May 2021. Data were analyzed independently in duplicate by thematic analysis. The Theory of Planned Behaviour (TPB) was the conceptual framework used to organize dominant themes.

**Results:**

We conducted a total of 60 individual interviews (137 eligible individuals contacted, 43.8% response rate) and identified six themes organized according to the three constructs of behavioural, normative and control beliefs as described in the TPB: (1) Behavioural: My “New Normal,” Individual Rights and Perceived Pandemic Severity, Fatigue with COVID-19, (2) Normative: COVID-19 Collective, (3) Control: Practicality of Public Health Guidelines, and (6) Conflicting Public Health Messages. Most (n = 43, 71.7%) participants perceived individuals in their geographic community to be following public health guidelines adequately. Several participants (n = 15, 25.0%) commented on the unequal impact of restrictions based on socioeconomic factors (i.e., class, race, age).

**Conclusion:**

Individual perceptions of risk, loss of control, access to resources (i.e., childcare), and societal expectations, shaped intentions to engage in disease preventative behaviours (i.e., social distancing) during the COVID-19 pandemic.

**Supplementary Information:**

The online version contains supplementary material available at 10.1186/s12889-023-15344-0.

## Introduction

Infectious disease outbreaks pose severe risks to public health [1, 2]. Targeted public health interventions (e.g., masking) are effective tools for mitigating transmission of viruses such as the severe acute respiratory syndrome coronavirus 2 (SARS-CoV-2), the virus which causes the coronavirus disease (COVID-19) [2–4]. Throughout the COVID-19 pandemic individuals have been required to alter their behaviour to adhere to public health guidelines such as physical distancing, masking, and restriction on indoor and outdoor gatherings—which were frequently updated or modified to reflect emerging science [5]. The influence of information related to COVID-19 (obtained through public health guidelines or through mainstream and social media) on behavioral intentions, is unknown. Therefore, understanding Canadians’ intentions to comply with public health measures, and the role that media plays in shaping these intentions, can provide policy stakeholders and decision makers with information to inform and support targeted messaging that promote compliance with public health safety measures.

A systematic review conducted prior to the COVID-19 pandemic found that perceived severity and susceptibility of disease are predictors of individual behaviour during periods of health crisis [6]. Past research has also found that an individual’s perceived efficacy of their behaviour in keeping them safe is linked to compliance with public health guidelines [6–8]. The Theory of Planned Behaviour (TPB) is a behavioural psychology model that connects beliefs to behaviour, including the factors that shape individual intentions to engage in certain behaviour [9–13]. In previous pandemics (SARS, H1N1), researchers reported a relationship between health behaviours in response to infectious disease outbreaks, and constructs of the TPB that included behavioural beliefs (*personal* attitudes toward behaviour), normative beliefs (subjective expectations of *others*), and control beliefs (beliefs that depend on *external* barriers) [9–11, 14, 15]. These results have been replicated within the context of the COVID-19 pandemic regarding intentions to receive vaccination [9, 11, 15]; however, little is known about beliefs that are associated with individual intentions to comply with the broader scope of public, non-pharmaceutical health guidelines (e.g., restrictions, masking, hand-washing) [6, 8, 10] and particularly, the role that access to and consumption of COVID-19 related information plays in shaping these intentions. Widespread adherence to public health guidelines is fundamental to preventing negative health and economic impacts as a result of the COVID-19 pandemic[16]. We used the TPB as the underpinning conceptual framework to develop an in-depth understanding of behavioural beliefs, normative beliefs, and control beliefs, and their influence on intentions to adhere to public health guidelines during the COVID-19 pandemic.

## Methods

### Study design

We employed a qualitative description study design [17] that was conducted in accordance with the Consolidated Criteria for Reporting Qualitative Research (Supplemental Table 1), a checklist developed to promote methodological rigor and transparent reporting of qualitative research[18]. This study was approved by the University of Calgary Conjoint Health Research Ethics (ID: REB20-0358) and Dalhousie University Health Science Research Ethics Board (ID: REB2020-5121). The use of a qualitative descriptive study design provided insight from a diverse group of individuals on perspectives and experiences regarding beliefs and intentions to adhere to public health guidelines during the COVID-19 pandemic. Semi-structured interviews for this study were conducted from February 11, 2021, to May 19, 2021.

### Participants

**Fig. 1:**
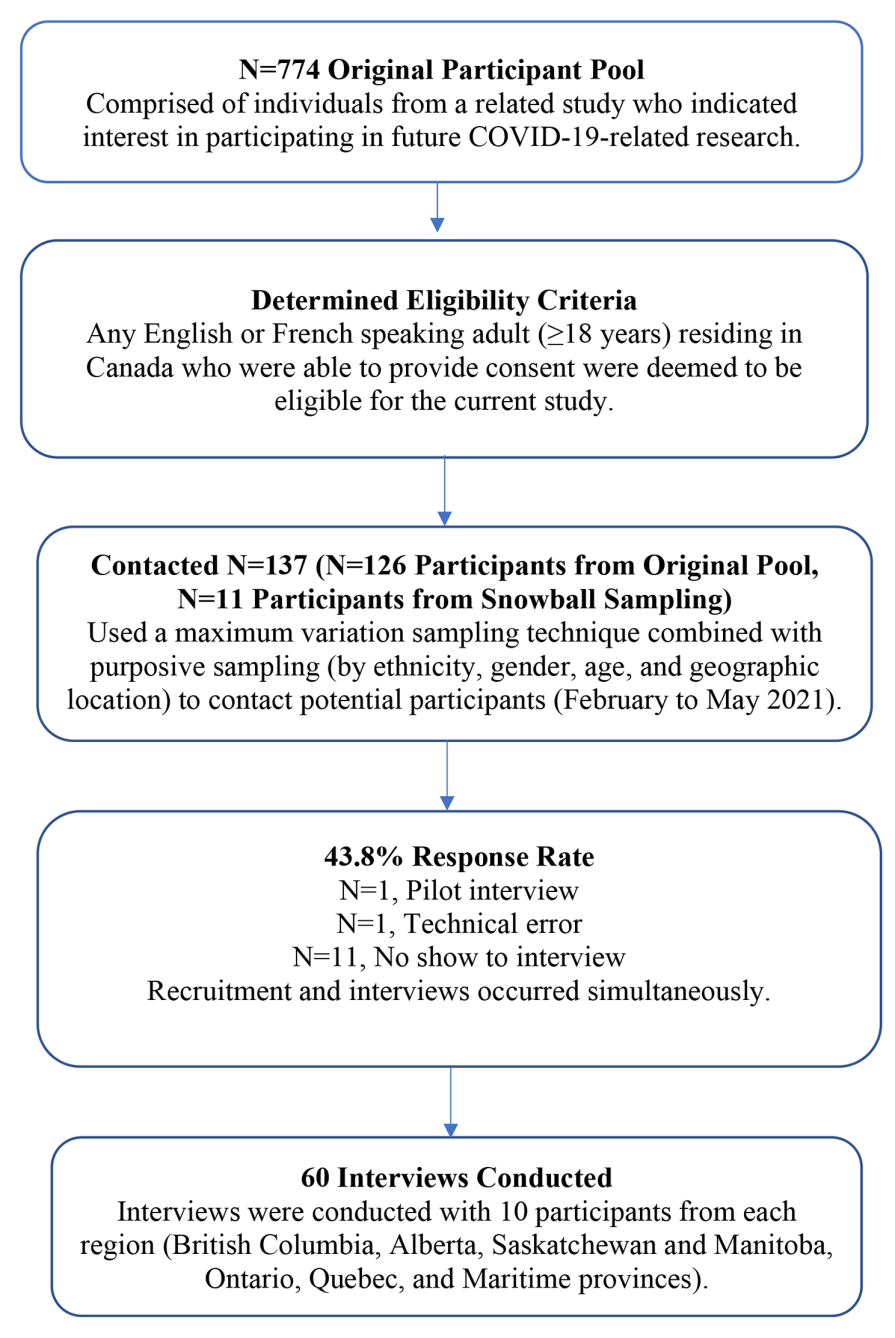
Participant Recruitment Flow Chart

Participants were initially recruited through a related study [19] where they had agreed to be contacted for future COVID-19 research. Figure 1 illustrates the participant recruitment process.

Individuals were eligible if they were English or French speaking adults (≥ 18 years of age), living in Canada, and able to provide informed consent. We used maximum variation sampling and snowball sampling to purposely recruit a target of 60 participants [20] diverse in gender, age, ethnicity, and geographic regions in Canada (Fig. 1). This target was driven by the aim to have 10 participants from each of the six regions (British Columbia, Alberta, Saskatchewan and Manitoba, Ontario, Quebec, and the Maritime Provinces). Diversity across participants was achieved by purposively sampling individuals based on broad categories of gender, age, ethnicity, and geographical region. Informed consent was provided by all participants prior to the start of the interview.

### Data collection

We iteratively developed a semi-structured interview guide to explore three broad topics with participants: (1) access and evaluation of information, (2) perspectives on mainstream and social media coverage of COVID-19, and (3) influences of messaging on individual and community perceptions of the pandemic and intentions to comply with public health measures (Supplemental Table 2). The interview guide was pilot tested with four members of the public for clarity and consistency with the study aim. Interviews were conducted in English and French by two researchers (SJMi, ML) experienced in qualitative methods; interviews occurred via Zoom (https://www.zoom.us/) or telephone, dependent on participant preference. The interview guide was developed to be 30 min; interviews lasted on average 27 min. Participants were asked 12 open-ended interview questions followed by 12 demographic questions at the conclusion of the interview. Interviews were digitally recorded to produce verbatim transcripts. English audio files were sent to a transcription company (www.rev.com/); French audio files were transcribed using NVivo 12 (QSR International, Melbourne, Australia), corrected by a fluent research team member (SJMi), and then translated by an artificial intelligence software (Sonix; https://sonix.ai/). French-to-English transcripts were reviewed a final time by the same fluent researcher (SJMi) to ensure accuracy. All textual data was reviewed, cleaned, and de-identified before analysis. Participants were given the chance to review their transcripts as a form of member-checking; however, none elected to do so.

### Data management and analysis

Data was managed and analyzed thematically using NVivo 12 (QSR International, Melbourne, Australia) [21]. Three researchers (SJMi, CD, EF) began analysis by reviewing and coding a random sample of transcripts (n = 5) independently, and in triplicate using open coding [22]. Initial codes were discussed with a senior qualitative researcher (JPL) to create the first draft of the codebook. Using open and axial coding [22], another 25 transcripts were analyzed by the same researchers, and the codebook was expanded and refined iteratively. The final codebook was applied to the complete dataset (n = 60 transcripts) that was coded independently, in duplicate (Supplemental Table 3). Researchers took continuous, detailed notes throughout coding and theme development. Researchers met weekly to discuss, revise, and refine themes that emerged from the data [21]. Themes were categorized into the TPB constructs (i.e., behavioural beliefs (one’s attitude toward the behaviour and the perceived outcome as positive or negative), normative beliefs (one’s perception of others’ beliefs about the behaviour), and control beliefs (the presence or absence of internal and external factors that impact the ability to perform the behaviour [23]).

**Table 1:** Interview participant characteristics (n = 60)

Characteristic	N (%)
**Region in Canada** ^1^	
Alberta	9 (15.0)
British Columbia	10 (16.7)
Maritimes^2^	11 (18.3)
Ontario	12 (20)
Québec	9 (15.0)
Saskatchewan/Manitoba	9 (15.0)
**Age category, years**	
Median, IQR	47.0 (34.5, 63.0)
18–29	9 (14.8)
30–44	21 (34.4)
45–64	17 (27.9)
65+	14 (23.0)
**Gender**	
Women	27 (45.0)
Men	33 (55.0)
**Ethnicity** ^3^	
White	38 (63.3)
Asian	14 (23.3)
Black	2 (3.3)
Latin American	1 (1.7)
Middle Eastern	1 (1.7)
Multiracial	3 (5.0)
**Education** ^1^	
Highschool	7 (12.1)
Some post-secondary	40 (69.0)
Post-secondary degree	11 (19.0)
**Household Income** ^1^	
$0-$50,000	15 (25.9)
$50,000-$99,999	30 (51.7)
$100,000 and over	13 (22.4)
**Employment Status** ^1^	
Full-time	31 (53.4)
Part-time	4 (6.9)
Retired	17 (29.3)
Other^4^	6 (10.3)
**Marital Status** ^1^	
Single	21 (36.2)
Partnered	27 (46.6)
Divorced/Widowed	10 (17.2)
**Has children** ^1^	
Yes	24 (41.4)

^1^ Missing data, n = 2

^2^ Maritimes region includes Nova Scotia, New Brunswick, and Prince Edward

^3^ Missing data, n = 1

^4^ Other includes unemployed, maternity leave, disability

## Results

We invited 137 individuals to participate (Fig. 1) and conducted a total of sixty individual interviews (43.8% response rate) (Fig. 1). Participants in this study were evenly distributed amongst six geographical regions with a median age of 47.0 (Table 1). Slightly over half of participants identified as male (n = 33, 55.0%) and most participants had completed some post-secondary education (n = 40, 69%). The majority (n = 43, 71.7%) of participants perceived individuals in their geographical community to be following public health guidelines for COVID-19 adequately. Some (n = 8, 13.3%) participants believed that age or ethnicity was connected to individual’s compliance with pandemic behaviors. Many (n = 15, 25.0%) participants commented on the unequal impact of restrictions based on socioeconomic factors (i.e., class, race, age) and some (n = 8, 13.3%) argued that tighter and more immediate restrictions were needed. When asked about where they obtained COVID-19 information, almost all (n = 51, 85.0%) participants mentioned the internet first. Television was also frequently noted (n = 43, 71.7%), as was the radio albeit less frequently (n = 17, 28.3%). Most participants from the Maritime Provinces praised their provincial government’s pandemic preparedness (n = 9, 81.8%), whereas participants from Ontario (n = 10, 83.3%) and Saskatchewan/Manitoba (n = 7, 77.8%) were often critical of their provincial government’s pandemic preparedness. We found no differences in beliefs according to age.

Thematic data analysis resulted in 6 themes within the TPB framework: *Behavioral Beliefs*: My “New Normal”, Individual Rights and Perceived Pandemic Severity, Fatigue with COVID-19; *Normative Beliefs*: COVID-19 Collective; *Control Beliefs*: Practicality of Public Health Guidelines and Conflicting Public Health Messages (Fig. 1).

### Behavioural beliefs

In this study, behavioural beliefs reflected individuals’ evaluation of how they’ve adapted to the current situation, as well as the behaviour of those around them, and the reasoning for why people behave the way they do. When reflecting upon the past year, participants described their acceptance—albeit reluctantly—of their “new normal,” which involved adjusting their daily lives to adhere to public health guidelines to prevent the spread of the virus. Many participants described frustration with others who were not actively complying to health guidelines, including conflicts that arose from what they perceived as clashing values systems. The dominant themes that emerged from these conversations included: individual rights and perceived pandemic severity, as well as fatigue with the COVID-19 pandemic generally.

#### My “new normal”

For most participants, there was an overwhelming belief that the COVID-19 pandemic was here to stay indefinitely. Participants clarified that they had accepted that adhering to public health guidelines was the reality of (their new) day-to-day life.

*“It’s around here forever now. In my opinion, … until there’s an actual cure and there won’t be a cure because of the variants … we’re going to have to get boosters on an annual basis just like the flu shot to keep the numbers down. … So this is our new normal.” 59-year-old female from Ontario*

In addition, participants described with tangible sadness how small daily joys had to be altered to adhere with public health guidelines. The eventuality of this shift was a shared belief for many.

*“We used to go out for coffee in the morning with a bunch of friends… We don’t do that anymore. Life has changed completely. I don’t think it’ll ever go back to where it was before the pandemic.” 75-year-old male from Alberta*

#### Individual rights and perceived pandemic severity

When evaluating the behaviour of others, including their decision to follow public health guidelines, participants had varying perspectives. Some participants directed feelings of anger towards people who (in their perspective) were not taking the pandemic seriously. These participants posited that a lack of compliance to guidelines was related to long-held beliefs that the pandemic did not pose as a serious health risk to Canadians.

*“Once again, it comes down to the fact that if you don’t think that this is a threat, you’re not going to follow anything. Doesn’t matter who’s directing you.” 62-year-old male from New Brunswick*

While many participants expressed incredulity towards those who prioritized individual freedom over the safety of the collective, some participants reflected on the influence that a loss of control could have on people’s sense of control and safety.

*“People don’t like not being in control of whatever the scenario may be. Doing something that helps them feel like they’re in control, gives them security and safety.” 36-year-old male from Ontario*

Another participant voiced concerns about government control regarding vaccine passports and access to personal health information.

*“I don’t want my health information on an app, you know what I mean? I don’t want anything ... I just feel it’s blurring the lines between government and your personal health details. I’m very uncomfortable, especially if big tech gets involved.” – 47-year-old female from Prince Edward Island*

Most participants commented on perceived pandemic severity, and relatedly, individuals’ selfishness. Older adults (particularly in Ontario and British Columbia) were more specific in who they perceived were selfish, pointing to younger adults as the “cause” of spread.

*“The people who need to be shaken and woken up, are the young people who probably don’t read the newspaper.... And maybe don’t even watch the TV news and unless they’re getting a blast on their cell phones, they don’t care. And that’s why they’re having [inaudible] parties and the most ridiculous things happening.” 81-year-old female from Ontario*

#### Fatigue with COVID-19

Many participants acknowledged the challenge of adhering to public health guidelines as the pandemic wore on, with physical distancing recommendations a particular point of contention.

*“I think in the beginning everybody was … following it [public health guidelines] as much as they possibly could because they realized that this was a serious thing .... But I think the more time that went on ... people are getting frustrated, really frustrated, and really upset. Anybody that I talk to is just annoyed with everything” 28-year-old female from Ontario*

Participants attributed COVID-19 fatigue to extended isolations, describing the toll that loneliness takes on people, psychologically.

*“I think people are just getting a little fed up that isolation, this isn’t the way to live. I mean, you can’t just stay holed up in your home forever and not see anybody because there’s a risk of something happening.” 63-year-old male from Manitoba*

While the majority of participants openly described fatigue in relation to others failure to comply with guidelines, few discussed fatigue in regard to their own behavior. Notably, when discussing COVID-19 fatigue, some participants clearly distinguished between “us,” and “them”.

*“I mean, at this point I think people are just getting tired. So, we’re seeing lots more of our ... People who aren’t in our inner circle of friends, that are starting to push the boundaries, and you’re like, “Come on guys, the end’s in sight. We just got to hold the line a little longer. Why are you starting to do things like that? Why are you going to social things?“ Things like that.”* – *40-year-old male from British Columbia*

### Normative beliefs

In this study, normative beliefs reflected the subjective norms held by participants, or their perceptions of what they think that others should or should not do, regarding a specific pandemic behaviour. This was most evident when participants discussed the importance of collective action and the influence this had on intentions to behave in accordance with public health guidelines.

#### COVID-19 collective

The majority of participants noted a sense of community action, describing that prioritizing community safety over their individual selves was the “right” thing to do. When providing examples of collective action in their own lives it was clear that participants were proud to be part of the collective placing the health of others over personal desires.

*“I think anytime something happens, the community comes together and they’re not necessarily focused on themselves. It’s more so for the community… Even just recently when we had back to back days [of high case numbers] the community came together and did their part [to get cases under control].” 27-year-old male from Nova Scotia*

Some participants recalled their skepticism surrounding collective action at the start of the pandemic. For these participants it was evident that the perceived behavioural expectations, set by the community, influenced their behavior as well as their intentions to behave.

*“To tell you the truth, in the beginning especially, I didn’t do any of it [follow public health guidelines] because it all wasn’t mandatory but then I went along and I wore the mask and I did all that stuff just to be courteous, to be respectful of other people who would be paranoid otherwise. As far as how I felt for myself, concerned? No, I wasn’t.” 65-year-old male from Alberta*

### Control beliefs

While participants were largely supportive of public health guidelines, their control beliefs were reflected in their concerns about people’s ability to comply with rules due to a lack of access to certain resources. Participants reflected on the practicality of public health guidelines, as well as conflicting messaging, commenting on their ability and intentions to adhere to these guidelines.

#### Practicality of public health guidelines

Many described that their intentions to engage in preventative behaviour was linked to the perceived practicality of public health guidelines. Most participants described restrictions as reasonable within the context of their own lives. Some participants voiced concern for vulnerable groups (i.e., older adults, adults without homes, single parents) acknowledging the added challenge to adhere to guidelines.

*“[people] are just unable to...for structural reasons…are unable to follow the recommendations out of necessity, because of the way they live… for example single women who go to work and then there is no one who can look after the children… they will not have the choice.” 39-year-old female from Quebec*

Finally, participants argued that preventative measures often fail to accurately represent the complexity of individual lives, and regularly discussed the challenges of trying to balance preventive measures with other health issues and needs.

*“I feel like with using the media, we’ve painted this brush of, “No other problems are going to exist other than the pandemic until the pandemic is over, so let’s just deal with the pandemic, and then we can get back to normal. Then, we’ll deal with your mental health problems, or then we’ll deal with your cystic acne or your bad digestion… We’ve cut back on all of these services, because right now the pandemic is the thing.“ I think that what’s been communicated to us, the overall message has kind of been diluted, I guess, or spun to make it like, “This is a problem of the individual person,“ and then a lot of people I know just carry around this intense and immense guilt for not doing well enough with the regulations.” 23-year-old female from Nova Scotia*

#### Conflicting public health messages

Participants unanimously agreed that individuals’ intentions and ability to follow public health guidelines depends fundamentally on their understanding of set guidelines. As healthcare in Canada is managed provincially, government responses to COVID-19 outbreaks varied. Participants described that this led to increasing confusion, coupled with the everchanging nature of public health guidelines and information.

*“As you know, in New Brunswick, we have colors [to delineate phases]. One color, nobody knew, wait now, wait now, wait now. What do we do here? … they’d say, “Well, yellow isn’t quite the same as yellow was last.“ That’s where we didn’t know. We didn’t know whether we were allowed to go to the hospital to visit people. We didn’t know anything. Couldn’t figure it out.” 82-year-old female from New Brunswick*

Participants deepened their discussion on confusion by describing how their intentions and willingness to engage in appropriate behaviour was influenced by lack of understanding appropriate behavior at any given time. While many participants acknowledged the challenge for health authorities and governments to stay on top of the science, the influence of contradictory messaging on people’s ability and intention to engage fully in preventative behaviour was recognized.

*“I think, like I say, they’re [public health officials] inconsistent. One day they say this, and the next day they say that ... I think people are just tuning out, quite frankly, because they’re frustrated, and because they’re not consistent.” 47-year-old female from PEI*

## Discussion

We conducted a qualitative description study using the TPB as the underpinning conceptual framework to investigate behavioural beliefs, normative beliefs, and control beliefs, and their influence on intentions to adhere to public health guidelines during the COVID-19 pandemic. Our findings from semi-structured interviews suggest that for behavioral beliefs, acceptance, perceived risks, and loss of control, shape participant’s intentions to behave. For normative beliefs, we found that adhering to protective behaviours is linked to collective action and perceptions of societal expectations. Finally, for control beliefs we determined that confusing pandemic health guidelines and contradictory messaging from regulatory bodies and news outlets influenced individuals’ ability to follow public health guidelines. Understanding the role that information plays in shaping these beliefs is critical, particularly as we grapple with a wealth of misinformation. For some, the information they received encouraged compliance by building confidence in the efficacy of health regulations. For others, this information sowed fear and increased confusion as contradictory messages appeared everywhere.

Our findings on intentions to behave in accordance with public health guidelines reflect earlier research regarding individuals’ fear, trust, and compliance generally within the context of global health crises. One study conducted in both Italy and France during the first wave of the COVID-19 pandemic found a positive correlation between the likelihood to sacrifice constitutional rights (i.e., freedom of peaceful assembly [24]) in the face of regulations given increasing concern about the virus [25]. While researchers have found that fear typically increases compliance [26], studies report the dangers of using fear to shape behaviour [27]. According to Witte’s extended parallel process model, when individuals believe that the recommended response to a threat is ineffective, or that they are unable to perform the action within (subjective) reason, they actively employ fear control responses such as denial or minimization [27]. This aligns with research reporting that behavioural compliance is associated with perceived effectiveness of the behaviour [11, 13, 14]. That our findings replicate and deepen prior research underscores the importance of ensuring that public health messaging is clear and consistent to promote confidence and widespread compliance to public health guidelines. A coordinated effort from provincial and federal leaders, along with the support of news media organizations, is crucial to achieving this.

The “collective” and the importance of engaging in pandemic preventive behaviours to protect their wider community was a common theme in our study. Within the context of the COVID-19 pandemic, research has highlighted how dominant language in the media generally supports the moralization of certain pandemic behaviour. For example, words like “covidiots” reinforces the idea that failure to comply with guidelines is attributed to lack of intelligence, common sense, or morality [28]. Relatedly, many participants in our study attributed a failure to comply with guidelines to selfishness, individualism, or ignorance. Other research has found that this may be associated with fear control responses, or a perceived lack of efficacy in the behaviour [11, 14, 15, 27]. Behavioural norms and ideas about morality can also gloss over the reality that differing behaviour reflects differing circumstances [29]. In line with other studies we found that the ability to comply fully with guidelines was tied to access to resources such as childcare, sick leave, and stable housing [29]. These findings also align with the TPB’s control beliefs, further emphasizing the importance of understanding how people’s socioeconomic location shapes their perceptions of health guidelines. Policy and decision makers should consider population-specific factors with the potential to influence intentions to adhere to guidelines.

### Limitations

There are limitations to consider when interpreting our findings. First, we were unable to contact potential participants from Newfoundland or the Territories as they were not surveyed in the previous study associated with this work. The absence of their perspectives and experiences in this work reflects a potential gap in our knowledge. Second, due to the recruitment methods, selection bias is possible as the consenting participants were individuals who had participated in a previous study, indicating an interest in COVID-19 research well before the interview took place. It is possible these individuals are more likely to follow health guidelines and continue to engage in pandemic preventive behaviour. Third, while our sample was diverse based on age, gender, and region, the qualitative nature of this study meant that findings could not be generalized statistically and applied to the broader population. Fourth, it is important to note that while several ethnic groups were present in our sample, the majority of participants were white-identifying. Future work should include an ethnically diverse sample in sufficient numbers to understand how culture and ethnicity might shape pandemic-related behaviour. Fifth, as the data collection took place online, perspectives from individuals who did not have access to, or sufficient understanding of the internet are potentially missing from this work. Sixth, the TPB has been used in quantitative studies as a framework for predictive modelling of behaviour [30]. While qualitative data prevents predictive modelling, the nature of our work allows for a nuanced understanding of the factors that influence behaviour in the COVID-19 pandemic. Lastly, the TPB is argued to have limitations (e.g., lack of environmental and economic considerations, assumes a linear decision-making process). We used the TPB as a guiding conceptual framework only and did not deductively develop our findings (e.g., codes, themes) into a pre-existing coding framework, which could have narrowed our potential findings. We inductively developed our findings to ensure they were rooted in the data and subsequently categorized the themes into the TPB to present easy and digestible findings situated within a well-established framework.

## Conclusion

This difficult period presents the unique opportunity to deepen our understanding of how to advance prevention-focused, population-level, public health guidelines. While the COVID-19 pandemic represents a unique threat, it is imperative that we recognize the late effects that are yet to come—human behaviour disorders, fear complexes, and dominant narratives about morality, that often emerge after health crises. Improving individuals’ knowledge, beliefs and behaviours toward preventative public health guidelines will improve overall public health during current and future public health crises. The lived experiences of participants in this work emphasize the importance of consistent, transparent, and accessible information so that individuals can successfully implement preventative behavior in their daily lives to mitigate the impact of the COVID-19 pandemic. Research must continue to examine adherence to public health guidelines to further understand how beliefs and intentions may shift over time.

## Electronic supplementary material

Below is the link to the electronic supplementary material.

Supplementary Material 1

## Data Availability

The datasets used and/or analysed during the current study are available from the corresponding author on reasonable request.
