# Supplementary material for: Exploring the influence of behavioural, normative and control beliefs on intentions to adhere to public health guidelines during the COVID-19 pandemic: a qualitative interview based study
Source: BMC Public Health. 2023 Mar 10;23:464. doi: 10.1186/s12889-023-15344-0 (PMC9999336; doi:10.1186/s12889-023-15344-0)
Supplement: Supplementary file 1 — Supplementary Material 1 [file 12889_2023_15344_MOESM1_ESM.docx]

**Contents**

[**Table 1.** Consolidated criteria for Reporting Qualitative research (COREQ) Checklist 2](#_Toc89849410)

[**Table 2.** Study Interview Guide 5](#_Toc89849411)

[**Table 3.** Coding Tree 9](#_Toc89849412)

# **Table 1.** Consolidated criteria for Reporting Qualitative research (COREQ) Checklist

| **Domain 1: Research team and reﬂexivity** | | |
| --- | --- | --- |
| *Personal Characteristics* | | *Location in Manuscript, section (page number)* |
| Which author/s conducted the interview or focus group? | SMi, ML | Page 4 |
| What were the researcher’s credentials? E.g. PhD, MD | Jeanna Parsons Leigh, PhD  Stephana J. Moss, PhD  Sara J. Mizen, MA  Emily FitzGerald, MSc  Rebecca Brundin-Mather, MSc  Chloe DeGrood, MSc  Alexandra Dodds, MSc  Henry T. Stelfox, PhD  Kirsten Fiest, PhD | Not reported in manuscript |
| What was their occupation at the time of the study? | Jeanna Parsons Leigh, Associate Professor  Stephana J. Moss, Senior Research Assistant and Team Lead  Sara J. Mizen, Research Assistant  Emily Fitzgerald, Research Assistant  Rebecca Brundin-Mather, Research Coordinator  Chloe DeGrood, Research Coordinator,  Alexandra Dodds, Research Assistant  Henry T. Stelfox, Professor, Intensivist  Kirsten Fiest, Assistant Professor | Not reported in manuscript |
| Was the researcher male or female? | Female: SJMi, EF, SJM, JPL, KMF, AC, RBM, CD  Male: HTS | Not reported in manuscript |
| What experience or training did the researcher have? | All (training in qualitative methods, facilitator experience) | Methods (page 4) |
| *Relationship with participants* | | |
| Was a relationship established prior to study commencement? | Yes | Methods (page 4) |
| What did the participants know about the researcher? e.g. personal goals, reasons for doing the research | Prior to commencement of the semi-structured interviews, participants were e-mailed the objectives of the semi-structured interview and understood it was a research project and ethical approval had been granted. Participants understood what their participation included, and all questions were answered prior to giving their consent. | Not reported in manuscript |
| What characteristics were reported about the interviewer/facilitator? e.g., Bias, assumptions, reasons and interests in the research topic | No interviewer-related biases identified. | Not reported in the manuscript |
| **Domain 2: Study design** | | |
| *Theoretical framework* | | |
| What methodological orientation was stated to underpin the study? e.g., grounded theory, discourse analysis, ethnography, phenomenology, content analysis | Qualitative Description study design (Kim, Sefcik and Bradway, 2017) | Methods (page 4) |
| *Participant Selection* | | |
| How were participants selected? e.g., purposive, convenience, consecutive, snowball | Using a maximum variation sampling technique | Methods (page 4) |
| How were participants approached? e.g., face-to-face, telephone, mail, email | Recruited via e-mail | Methods (page 4) |
| How many participants were in the study? | 60 | Table 1 (page 5) |
| How many people refused to participate or dropped out? Reasons? | 11 people did not show to interview | Figure 1 (page 15) |
| *Setting* | | |
| Where was the data collected? e.g., home, clinic, workplace | Virtually via phone or Zoom | Methods (page 4) |
| Was anyone else present besides the participants and researchers? | No | NA |
| What are the important characteristics of the sample? e.g., demographic data, date | Demographic data | Table 1, Results (page 5) |
| *Data collection* | | |
| Were questions, prompts, guides provided by the authors? Was it pilot tested? | Interview guides were not provided to participants but were pilot tested with research contacts outside of the team | Methods (page 4), Supplementary files |
| Were repeat interviews carried out? If yes, how many? | No | NA |
| Did the research use audio or visual recording to collect the data? | All semi-structured interviews were audio-recorded using an audio tape recorder | Methods (page 4) |
| Were field notes made during and/or after the interview or focus group? | Yes, but notes were not used in the data analysis | Not reported in manuscript |
| What was the duration of the interviews or focus group? | Interviews were between 20-30 minutes | Methods (page 4) |
| Was data saturation discussed? | Yes | Methods (page 4) |
| Were transcripts returned to participants for comment and/or correction? | No. Participants were told that they were able to request a copy of their transcript for review, but no participants reached out about this | Methods (page 4) |
| **Domain 3: analysis and findings** | | |
| *Data analysis* | | |
| How many data coders coded the data? | Three | Methods (page 4) |
| Did authors provide a description of the coding tree? | Yes | Supplementary Table 3 |
| Were themes identified in advance or derived from the data? | Themes were derived from the data | Methods (page 4) |
| What software, if applicable, was used to manage the data? | NVivo12 | Methods (page 4) |
| Did participants provide feedback on the findings? | No participants asked to review their transcripts. | Methods (page 4) |
| *Reporting* | | |
| Were participant quotations presented to illustrate the themes/findings? Was each quotation identified? e.g., participant number | Yes, quotes were identified by participant number, age, gender and region. | Results (pages 6-10) |
| Was there consistency between the data presented and the findings? | Yes | Results (pages 6-10) |
| Were major themes clearly presented in the findings? | Yes | Results (pages 6-10) |
| Is there a description of diverse cases or discussion of minor themes? | Yes | Results (pages 6-10) |

# **Table 2.** Study Interview Guide

| **Introduction** |
| --- |
| Thank you for agreeing to speak with us today. My name is [insert name] and I am a [study coordinator/research assistant] at Dalhousie University working on this study. We appreciate your participation in our national survey from April of last year, and also agreeing to participate in this follow-up study.  We are conducting interviews with individuals across Canada in order to better understand what Canadians think about the information related to COVID-19 that they are seeing, reading, or hearing, including prominent messages and their impact on perceptions and behaviors. We look forward to the opportunity to learn from your experiences and insights. The topics we will cover in this interview serve only as a guide. If there are other insights you would like to offer during the interview, we would like to hear them.  We emailed you a copy of the consent form which is part of the process of informed consent. It should give you a basic idea of what the research is about and what your participation will involve. Have you reviewed the consent form? Do you understand the conditions for participation and your rights? Do you have any questions about the informed consent form?  I do want to remind you that participation in this interview is completely voluntary. If at any point you feel uncomfortable with the process and wish to end your participation you are free to do so. If you would like to skip a question or end the interview early, feel free to let me know. If you do wish to withdraw your data, you will have 1 week following the interview to do so after which point it will no longer be possible to remove your data from the larger dataset. Do you consent to participating in this interview?  Do you have any questions?  Before we start, I would like to remind you that we will be recording this interview in order for us to accurately capture our conversation. Do you agree to be recorded for research purposes?  <If participant has provided informed consent, start recording>  Great. Thank you for participating. |
| 1. **Information Access** |
| - 1. Where do you primarily see information about COVID-19?   PROBES (If not incorporated into response to Q1.1)   - Can you tell me how you usually get your information about COVID-19?   [this may include active seeking, active scanning, or non-directed through a variety of general sources like TV, newspaper, twitter, conversations with family, etc]  (Alternative phrasing: If you were looking for information related to COVID-19, what would you do?)   - Have you changed how you get your information over the course of the pandemic (e.g., sources, search strategies)? [If yes, Why/How? If no, why not?]   1. Can you describe the kinds of information related to COVID-19 or the pandemic that you have looked for and why? PROBES - Topics: public health guidelines; infection rates; Vaccine development and rollout; Social Services access - Is it difficult to find the kinds of information that you are looking for? Challenges? Or not? |
| 1. **Evaluation of Information** |
| - 1. How do you evaluate the information about COVID-19 that you see, read, or hear? PROBES      - What does ‘credible’ information about COVID-19 mean to you? [how do you recognize false or misleading information?]      - Can you tell me about any information about COVID-19 that you saw, read, or heard that you thought was false or misleading?      - How do you think false or misleading information should be managed?   2. Are there certain kinds of information (formats or sources) that you avoid when looking for information about COVID-19? Why these ones? Or why not?   PROBES   - - - Conversely, are there certain sources that you consistently use? Why? |
| 1. **Media Coverage** |
| - 1. What do you think about the way the COVID-19 pandemic has been covered in the media, such as in print, online, or television?   PROBES   - Do you think the coverage been accurate, effective, transparent, etc…    1. Do you think media coverage (integrity of information) has changed over the course of the pandemic?   PROBES   - Do you find that you respond differently to coverage about COVID-19 since the start of the pandemic? - Are there messages in the coverage of COVID-19 that you’ve seen, read, or heard that stuck with you (resonated with you) (positively or negatively) – If yes, what and why; If no, why do you think that is? |
| 1. **Personal Impact of Messaging on Behaviours and Perceptions of the Pandemic** |
| - 1. How does the information that you have read or seen inform the decisions that you make in your everyday life (Alternative phrasing: Tell me about some of the ways that you have used information that you’ve seen, read, or heard about COVID-19 to inform decisions that you make in everyday life?)   PROBES   - Examples of information/coverage topics: public health guidelines; infection rates; vaccine development and rollout; social services access - Examples of everyday life decisions: going to the grocery store, restaurants, about sending your child to school, healthcare appointments, visiting family or friends, vaccination] - How has this changed over the course of the pandemic, if at all?   1. How has the communication of information about the COVID-19 vaccine and rollout impacted your decision on whether to be vaccinated or not?   PROBES   - Where are you getting your information and how are you using it? - Are you having to make decisions for other members of your family on whether to vaccinate or not? Yes/No. If yes, do you access and evaluate information differently to make this decision, then for yourself? |
| 1. **Community Impacts of COVID-19** |
| - 1. How would you evaluate your province’s response to the COVID-19 pandemic? PROBES - How would you compare your province’s response to other provinces/regions? [better, worse]   1. Thinking more locally, how do you think people in your community generally responded to public health guidelines?   2. Do you think the public health guidelines were clearly communicated to people? Were they reasonable/feasible to for everyone to follow? PROBES - Why do you think people fail to follow public health guidelines or rules? - Examples of guidelines: mask policies, travel restrictions, quarantine rules, lockdowns, etc. |
| **Closing Question** |
| What is the one thing you think people need to understand most about COVID-19 and the pandemic? How do you think the main media sources can help with this? [In what way? Or Why not?]  This has been a very good conversation. Is there anything else you would like to add / say related to media coverage of COVID-19?  Thank you for participating in our interview. <Stop Recording> |
| **Structured Demographic Questions** |
| We are collecting personal and family demographic information in order to describe our participants in aggregate. Contact information is only for us if you would like to review the report generated from this work to ensure that it reflects your experiences. Please note that your demographic information and contact info will be stored in a password protected database that is only accessible to the study research team. If you are not comfortable answering any of the below questions you are welcome to skip any questions that you do not wish to answer or decline answering any of the questions.  If applicable: What email address/mailing address do you wish to receive your transcript?   1. What province do you live in? 2. Do you live in a rural or urban area? 3. What is your age? 4. What gender do you most closely self-identify with? 5. What ethnic or racial do you most closely self-identify with? 6. Do you self-identify as Indigenous, First Nations, Inuk/Inuit, and/or Metis? 7. What is your highest level of education? 8. How many individuals live in your household? 9. What is your marital status? 10. Do you have any children? 11. Do your children live with you? 12. What is your child(ren)’s age(s)? 13. What is your employment status? (full-time/part-time/unemployed) 14. What is your household income (below $50K, between $50K and $100K, or >$100K) 15. What language do you speak most often at home? |
| **Gift card Information** |
| As a thank you for their participation, we give our participants a $50 e-gift card. Do you have a preference between:   - Chapters/Indigo - Starbucks - Tim Horton - Amazon   If participant would prefer another store, we can accommodate if reasonable. |

| **Table 3.** Coding Tree   \| Coding Tree \| \| --- \| \| Evaluating Information \| \| Contains data \| \| Cross-checking with multiple sources \| \| Ignore false information \| \| Judgement Based, Trust \| \| Trust in Source \| \| Information Seeking \| \| Active \| \| Internet \| \| Government Websites \| \| Change of Approach \| \| No Change \| \| Mixed Approach \| \| Passive \| \| Social Media \| \| TV News \| \| Information Source \| \| Middle Age \| \| HCP or Personal Contact \| \| Internet \| \| Podcasts \| \| Print \| \| Radio \| \| Social Media \| \| TV \| \| Older \| \| HCP or Personal Contact \| \| Internet \| \| Podcasts \| \| Print \| \| Radio \| \| Social Media \| \| TV \| \| Young \| \| HCP or Personal Contact \| \| Internet \| \| Podcasts \| \| Print \| \| Radio \| \| Social Media \| \| TV \| \| Media Coverage of COVID19 \| \| Access to Information \| \| Changes in coverage over time \| \| Consistent Throughout \| \| Impactful Stories \| \| Negative \| \| Confusing \| \| Deflection \| \| Fear mongering or sensationalism \| \| Inaccurate \| \| Lack of Transparency \| \| Overwhelming \| \| Repetitious \| \| Positive \| \| Accurate \| \| Reasonable \| \| Transparent \| \| Provincial differences \| \| Suggestions for Improvement \| \| Misinformation \| \| Examples of Misinformation \| \| Management of Misinformation \| \| Scam and rumours \| \| Mistrust \| \| American Media \| \| Local Media \| \| Provincial Government \| \| Lack of Transparency \| \| Social Media \| \| TV Media \| \| WHO \| \| Behaviour Change \| \| Community Response to COVID19 \| \| Mixed Response \| \| People Not Following the Rules \| \| Burn Out or Impatience \| \| Conscious choice \| \| Confusion \| \| COVID-19 Denial \| \| Peer Influence \| \| People not taking it seriously \| \| Religion \| \| Selfishness or Individualism \| \| Trust \| \| Racism \| \| Responded Well \| \| Health Guidelines and Rules \| \| Negative \| \| Confusion Regarding Rules \| \| Positive \| \| Well Communicated \| \| Public response to health guidelines \| \| Everyone just needs to follow the rules \| \| Rules not Feasible \| \| Should have had more rules \| \| Impact of COVID-19 on Individual \| \| Acceptance \| \| Employment and Income \| \| Family \| \| Personal Feelings about COVID19 \| \| Blaming certain groups \| \| Burnout \| \| Struggling with Unknowns \| \| Services (CERB, social assistance) \| \| Social Networks \| \| Impact of COVID-19 on Society \| \| Certain groups are more affected \| \| No change in information and behaviours over pandemic \| \| Politicization of COVID info and response \| \| What people need to understand most about COVID19 \| \| Get Vaccinated \| \| How the system has failed \| \| It is Serious \| \| Need to Work Together \| \| New normal \| \| Prepare for Next Disease \| \| Federal Government Response \| \| Good \| \| Poor \| \| Provincial Government's Response \| \| Alberta \| \| Good \| \| Poor \| \| BC \| \| Good \| \| Poor \| \| Maritimes \| \| Good \| \| Poor \| \| Ontario \| \| Good \| \| Poor \| \| Prairies \| \| Good \| \| Poor \| \| Quebec \| \| Good \| \| Poor \| |
| --- | --- | --- | --- | --- | --- | --- | --- | --- | --- | --- | --- | --- | --- | --- | --- | --- | --- | --- | --- | --- | --- | --- | --- | --- | --- | --- | --- | --- | --- | --- | --- | --- | --- | --- | --- | --- | --- | --- | --- | --- | --- | --- | --- | --- | --- | --- | --- | --- | --- | --- | --- | --- | --- | --- | --- | --- | --- | --- | --- | --- | --- | --- | --- | --- | --- | --- | --- | --- | --- | --- | --- | --- | --- | --- | --- | --- | --- | --- | --- | --- | --- | --- | --- | --- | --- | --- | --- | --- | --- | --- | --- | --- | --- | --- | --- | --- | --- | --- | --- | --- | --- | --- | --- | --- | --- | --- | --- | --- | --- | --- | --- | --- | --- | --- | --- | --- | --- | --- | --- | --- | --- | --- | --- | --- | --- | --- | --- | --- | --- | --- | --- | --- | --- | --- | --- | --- | --- | --- | --- | --- |
